# High Efficiency Dye-sensitized Solar Cells Constructed with Composites of TiO_2_ and the Hot-bubbling Synthesized Ultra-Small SnO_2_ Nanocrystals

**DOI:** 10.1038/srep19390

**Published:** 2016-01-13

**Authors:** Xiaoli Mao, Ru Zhou, Shouwei Zhang, Liping Ding, Lei Wan, Shengxian Qin, Zhesheng Chen, Jinzhang Xu, Shiding Miao

**Affiliations:** 1 School of Electronic Science and Applied Physics, Hefei University of Technology (HFUT), Hefei 230009, China; 2 School of Chemistry and Chemical Engineering, HFUT, Hefei, 230009, China; 3 Institut de Minéralogie, de Physique des Matériaux, et de Cosmochimie (IMPMC), Sorbonne Universités—UPMC Univ. Paris 06, Paris 75005, France

## Abstract

An efficient photo-anode for the dye-sensitized solar cells (DSSCs) should have features of high loading of dye molecules, favorable band alignments and good efficiency in electron transport. Herein, the 3.4 nm-sized SnO_2_ nanocrystals (NCs) of high crystallinity, synthesized via the hot-bubbling method, were incorporated with the commercial TiO_2_ (P25) particles to fabricate the photo-anodes. The optimal percentage of the doped SnO_2_ NCs was found at ~7.5% (SnO_2_/TiO_2_, *w*/*w*), and the fabricated DSSC delivers a power conversion efficiency up to 6.7%, which is 1.52 times of the P25 based DSSCs. The ultra-small SnO_2_ NCs offer three benefits, (1) the incorporation of SnO_2_ NCs enlarges surface areas of the photo-anode films, and higher dye-loading amounts were achieved; (2) the high charge mobility provided by SnO_2_ was confirmed to accelerate the electron transport, and the photo-electron recombination was suppressed by the highly-crystallized NCs; (3) the conduction band minimum (CBM) of the SnO_2_ NCs was uplifted due to the quantum size effects, and this was found to alleviate the decrement in the open-circuit voltage. This work highlights great contributions of the SnO_2_ NCs to the improvement of the photovoltaic performances in the DSSCs.

Dye-sensitized solar cells (DSSCs) based on semiconductor electrodes are of great interest as alternatives to the conventional silicon based solar cells in view of the ease of fabrication, cost-effectiveness and environmental benignancy^1^,^2^,^3^. An ideal photo-anode for DSSCs should combine features of high specific surface areas, fast electron transport and less interfacial electron recombination^3^,^4^. Intensive work has been devoted to the fabrication of TiO_2_ photo-anodes^5^,^6^. However, the TiO_2_ based anodes suffer from sluggish electron mobility and high density of surface states which gave rise to the charge recombination. To explore materials for more efficient photo-anodes, efforts have been made to utilize metal oxides such as ZnO, SnO_2_, Nb_2_O_5_^7^,^8^,^9^,^10^,^11^,^12^, and bi-functional materials including ZnO/TiO_2_, SnO_2_/TiO_2_, ZnO/SnO_2_, SrTiO_3_/TiO_2_, *et al*.^13^,^14^,^15^,^16^,^17^,^18^. Particularly, SnO_2_ has attracted great attention due to the two remarkable advantages: (1) SnO_2_ possesses a high electron mobility (100–500 cm^2^ V^−1^ S^−1^), two orders of magnitude higher than that of TiO_2_ (0.1–10 cm^2^ V^−1^ S^−1^), which would give rise to improved charge transfer^19^; (2) Compared to TiO_2_, SnO_2_ has a larger band gap of 3.6 eV and a more negative conduction band minimum (CBM = −4.56 V vs. vacuum), which would facilitate the electron injection from the sensitizer to the semiconductor electrodes^20^. However, the efficiency of SnO_2_ based DSSCs is still low up to date^21^. The low open-circuit voltage (*V*_OC_) was thought as reasoned by the more negative position of CBM^22^. The sluggish photo-to-electron efficiency (η) was mainly caused by the charge recombination which is usually trapped by the surface states^23^. After a thorough survey of literatures, we found that almost all of the reported photo-anodes based on SnO_2_ have barely considered the quantum-size effects. Nevertheless, this could possibly alleviate the decrement in *V*_OC_^24^,^25^. Herein, we propose our strategy to satisfy the following two requirements: (1) to improve the *V*_OC_, the level of CBM or the flat band potential (*V*_fb_) of the semiconductor anodes can be lifted by decreasing the sizes of SnO_2_ particles^26^; (2) to eliminate or alleviate the charge recombination at the interface, and thus it is desirable to prepare highly crystalline SnO_2_ nanoparticles^27^,^28^.

Although there have been a good number reports on preparing SnO_2_ particles, the previous authors always adopted a hydrothermal way^16^. Review papers can be found as some of the references^29^,^30^. Taking into consideration of the above two criterions, the hydrothermally method lacks in yielding high crystallinity because the reaction temperature is confined by the boiling point of water (~100 °C). The average sizes of the SnO_2_ particles were always difficult to be controlled. Therefore, it is necessary to find strategies to synthesize highly crystalline SnO_2_ plus uniform size-distributed nanocrystals (NCs). As suggested in our previous report^31^, the hot-bubbling synthesis, which was conducted by bubbling air into surfactant solutions dissolved with Sn-precursors, yields SnO_2_ NCs of high crystallinity and desired sizes^31^,^32^. Because the exciton Bohr radius (~3.0 nm) of SnO_2_ is much smaller than other semiconductors, the size effects of SnO_2_ are not easily observed^33^. In this research, we utilize this hot-bubbling method to get ultra-small SnO_2_ NCs (average size 3.4 nm), and incorporate these NCs in the TiO_2_ photo-anode to construct the DSSCs. Due to the high temperature performed in the synthesis and the fast diffusion rate provided by the gas reactants, homogeneous nucleation was achieved. The colloidal SnO_2_ NCs were found not to be aggregated during the annealing processes because of the well-protection by the ligand molecules. The SnO_2_ NCs were found not only to enlarge the surface area of the semiconductor anodes, but also to facilitate the charge transfer across the DSSCs. The SnO_2_/TiO_2_ composite films are demonstrated to be efficient anodes to boost the photovoltaic performances owning to the increased dye loading, the facilitated electron injection and the efficient charge collection.

## Results and Discussion

### Structural characterization of the SnO_2_ NCs

**Figure 1:**
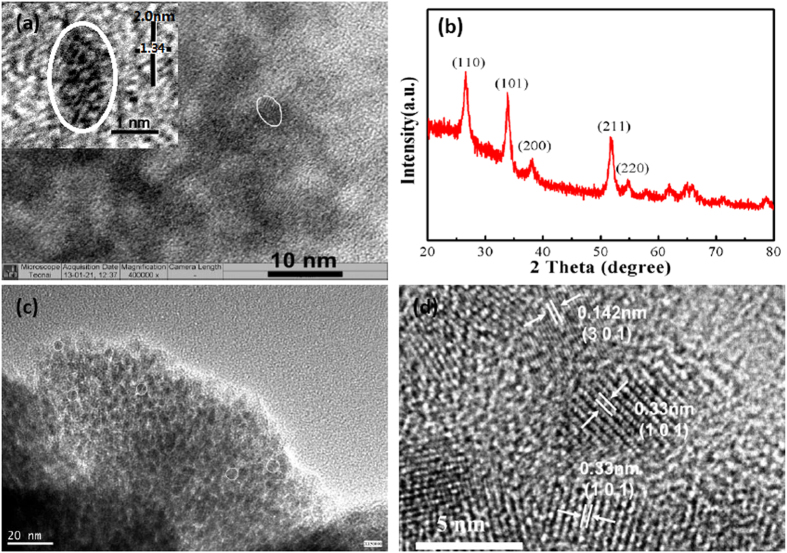
(**a**) TEM and HRTEM (inset) images of the colloidal SnO_2_ NCs synthesized via the hot-bubbling method; (**b**) XRD pattern of the colloidal SnO_2_ NCs; (**c,d**) The TEM (**c**) and HRTEM (**d**) images of the air-annealed SnO_2_ NCs (T = 450 °C, t = 2.0 hrs).

For a brief introduction of the hot-bubbling synthesis, the Sn-oleate was prepared from precursors of oleic acid and the newly prepared SnO·xH_2_O in 1-octadecene (ODE) solutions at temperatures of 280~320 °C. When a flow of room-temperature air was bubbled into these hot solutions, an explosive nucleation in form of SnO_2_ clusters occurs in the O_2_-induced hydrolysis reactions^31^. The high diffusion rate of the gas benefits the fast nucleation. The air flow cools down the micro-environment of the SnO_2_ clusters, which would avert the Oswald ripening growth^34^. Therefore, the hot-bubbling syntheses yield homogeneous clusters of high crystallinity. Herein we observed that the as-prepared colloidal clusters were of ellipse shapes, and assembled into network structures. A typical TEM image of the as-synthesized sample is shown in Fig. 1a. The inset is a high-resolution TEM (HRTEM) image of a single particle. It was found that the long axis of the ellipse particle was ~2.0 nm, and the short axis was ~1.34 nm. Only several periodic lattice fringes were observed across this particle. Sizes of this range are equivalent to the Bohr radius of the exciton of SnO_2_^33^, which makes these clusters show prominent size effects. The quantum size effects can be reflected by our previous measurements on UV-vis absorption spectroscopy^31^. The clusters could not grow into huge particles even the growth time was prolonged (e.g., 30.0 min). This suggests that the growth of SnO_2_ is encountered with a high energy barrier in this OA-ODE-OLA hot solutions. The XRD pattern of the colloidal sample, shown in Fig. 1b, reveals that the tetragonal cassiterite type of SnO_2_ (JCPDS no. 41–1445) was synthesized^16^. Figure 1c displays a TEM image of the neat SnO_2_ NC annealed in air at the temperature of 450 °C. Semi-spherical particles were observed, and the diameter ranges from 3.0 to 4.0 nm. The average size (3.4 nm) of the annealed particles was evaluated by the Scherrer equation^35^. We thus named the hot-bubbling synthesized sample as the 3.4 nm-SnO_2_ NCs. The HRTEM image is shown in Fig. 1d. Well-resolved lattice fringes were found, and this suggests the high-crystallinity of the SnO_2_ particles. The distances of the fringes are estimated to be 0.142 and 0.330 nm, which can be well indexed to the *d*-spacing correlated with the (301) and (101) of the tetragonal SnO_2_. In contrast, the commonly used hydrothermal synthesis yields SnO_2_ particles of a spherical shape^22^, and the average diameter is about 20 nm. We name this sample as S_20 for a control study.

### Morphological studies on the fabricated SnO_2_/TiO_2_ films

**Figure 2:**
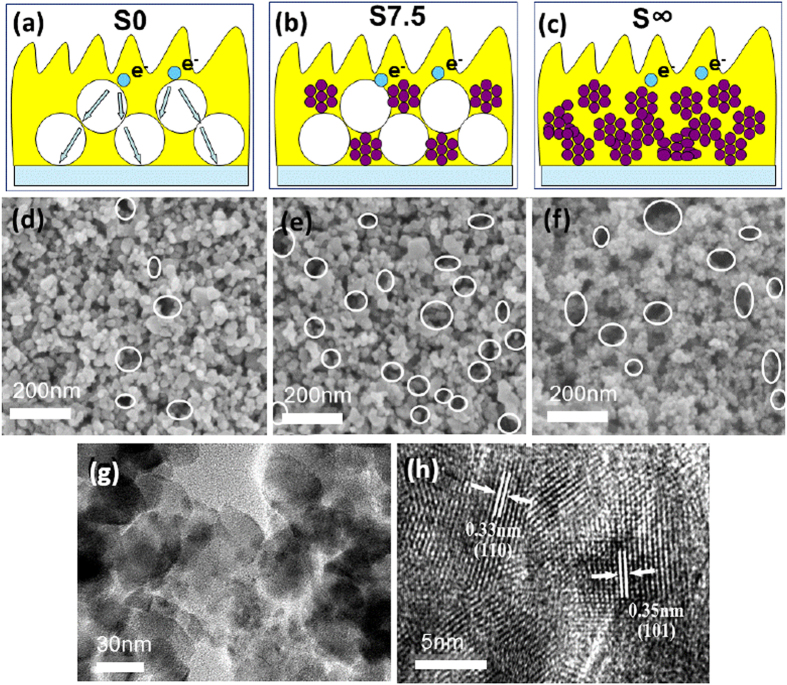
(**a–f**) Schematic presentations and SEM images of the SnO_2_/TiO_2_ photo-anodes prepared with the percentage of 0% (**a,d**), 7.5% (**b,e**) and ∞% (**c,f**) SnO_2_; (**g,h**) TEM images for S7.5 at different magnifications.

The above annealed SnO_2_ were mixed with the commercial TiO_2_ particles (P25, D = ~25 nm) to fabricate photo-anodes for the DSSCs. Figure 2a–c show schematic diagrams for the SnO_2_/TiO_2_ photo-anodes prepared by using different ratios of SnO_2_/TiO_2_. The possible pathways followed by the electron transfer are also demonstrated, which is obtained from the following analyses. Percentages of SnO_2_/TiO_2_ = 0%, 7.5% and ∞% (*wt.*) are denoted, respectively in Fig. 2a–c. We named these films as S0, S7.5 and S∞. The data in the sample name means the percentage of SnO_2_ doped with respect to TiO_2_. For example, the sample of S0 denotes the anode films were made with pure TiO_2_ particles while the film S∞ comprised totally with the SnO_2_ NCs. The SEM images for films S0, S7.5 and S∞ were shown in Fig. 2d–f. The porous structures were clearly found in these anodes. Specifically the composite film S7.5 showed more porosity with tiny holes, and these holes (shown as circles) are uniformly distributed in the film (Fig. 2e). Although the SEM image can only give views in a micrometre sized area, the smaller but uniform distribution can be further confirmed by the N_2_ adsorption-desorption characterizations. Herein, it is easy to understand that the mixed particles of different size levels (D_SnO2_ = 3.4 nm, D_TiO2_ = 25 nm) provide more chances for particles approaching, and usually assemble into networks in the photo-anodes^36^. Thus we might deduce that paths for electrons transport change from point-to-point in the pure TiO_2_ films to point-to-surface or surface-to-surface in the composite SnO_2_/TiO_2_ films^25^. Closer contact of the semiconductor oxides is expected to accelerate the charge transfer in the photo-anodes. The TEM images (Fig. 2g) illustrates that the SnO_2_ NCs are well dispersed in the composite films. The typical HRTEM image in Fig. 2h shows the close packing of TiO_2_ and SnO_2_ NCs (see the labelled *d*-spacing of TiO_2_ and SnO_2_), which further confirms that the ultra-small SnO_2_ tends to be incorporated and assembled uniformly among the TiO_2_ particles.

### Characterizations on the SnO_2_/TiO_2_ films

**Figure 3:**
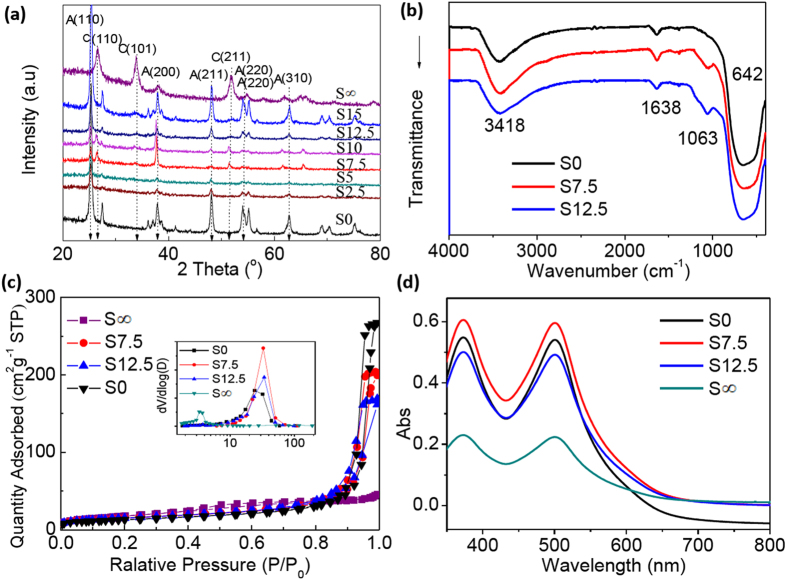
(**a**) The XRD patterns of powders scraped from the photo-anode films that are incorporated with different ratios of SnO_2_ NCs (S0-S12.5); (**b**) The FT-IR spectra for the SnO_2_/TiO_2_ films (S0, S7.5 and S12.5); (**c**) N_2_ adsorption-desorption isotherms of the SnO_2_/TiO_2_ films, and the inset shows the corresponding BJH pore size distribution plots; (**d**) The UV-vis spectra of N719 desorbed from pre-fabricated photo-anode films of SnO_2_/TiO_2_ (S0, S7.5, S12.5 and S∞) using 0.1 M KOH.

The incorporation of SnO_2_ NCs in the composite photo-anodes were further studied by means of XRD, FT-IR, N_2_ adsorption-desorption and the electrical resistivity measurements. Figure 3a shows the XRD profiles of the fabricated films in which different ratios of SnO_2_ were incorporated (S0 → S∞). The strong diffraction peaks can be assigned to the anatase TiO_2_ (JCPDS No. 84–1285, labelled as ‘A’). We also found weaker peaks (110), (101) and (211) attributing to the cassiterite type SnO_2_ in films of S2.5–S12.5 (labelled as ‘C’)^37^. Figure 3b displays the FT-IR spectra of the composite films with three typical ratios of SnO_2_/TiO_2_ (S0, S7.5 and S12.5). The broad bands in range of 400–800 cm^−1^ are the stretching and bending modes of Ti-O-Ti^38^. The band at 1063 cm^−1^ observed in sample S7.5 and S12.5 can be attributed to the stretching vibrations of Sn-O-Sn demonstrating the presence of SnO_2_^38^. The strongest intensity of this band in sample S12.5 suggests uniform incorporation of SnO_2_. Figure 3c shows the N_2_ adsorption-desorption isotherms (type II hysteresis loop), and the derived data such as surface area, pore volume and size distribution are summarized in Table 1^16^,^39^,^40^. The Barrett-Joyner-Halenda (BJH) pore size diagram is plotted as an inset in Fig. 3c. We found a peak representing smaller sizes in the either pure SnO_2_ or composites of TiO_2_ and SnO_2_, yet the pure TiO_2_ has no such pores. This means the ultra-small SnO_2_ NCs produce smaller pores as well as enlarge the surface area of the composite. Or we can say the incorporation of SnO_2_ NCs would cause rougher surface of the TiO_2_ films. This would surely benefit a higher loading of dye molecules (N719). To study the saturated amount of N719 that are adsorbed by the composite, we recorded the UV-vis absorption spectra of the N719 solutions. The solutions were obtained by desorbing from a pre-fabricated photo-anode with the aid of KOH. The concentration of N719 was evaluated by recording the absorbance at a particular wavelength (λ = 500 nm)^27^. The data were also summarized in Table 1 (the 3^rd^ column). The sample S7.5 has the highest amount of N719. It was indicated that the adsorption capacity of dye molecules are related to the surface areas and pore volumes. As expected the incorporation of SnO_2_ NCs was found to increase the loading amount of N719 molecules. However, excessive incorporation of SnO_2_ would block the pores, and less amount of N719 molecules are immobilized (see samples S12.5 or higher ratios of SnO_2_/TiO_2_). Therefore, a certain amount of SnO_2_ should be incorporated in order to get a higher loading of N719. We also studied the conductivity of the fabricated films or the sheet resistance, and these measurements were conducted by means of the 4-point probe method. The Hall-effects were also evaluated. The collected data (Table 2) reveals that the resistivity of the SnO_2_/TiO_2_ composite films decreases with incorporation of SnO_2_ NCs, while the Hall mobility of charge carriers increases significantly. For example, the resistivity of the pure P25 film (S0) is 4.57 Ω cm, and such a value decreases to 3.74 × 10^−2^ Ω cm in sample S7.5. The charge carrier concentration was found to increase enormously (3 ~ 5 orders of magnitude) after the incorporation of SnO_2_. It is interesting to note that the carrier concentration is the highest (2.57 × 10^19^ cm^−3^) when the incorporation ratio of SnO_2_ is 7.5%, which is even higher than the pure SnO_2_. This suggests a synergistic effect might occur between TiO_2_ and SnO_2_, and this would provide advantages in enhancing the diffusion of charge carriers. On condition that measurements performed under the same lights and temperatures the mobility or concentration of charge carriers in bulk semiconductors is mainly determined by the band-gaps and Fermi levels^41^. Herein, we attributed the improvement to a shift of CBM due to the incorporation of SnO_2_, and the Fermi level in the composite is altered. Although the particle size is another factor to hinder the charge carries transport^42^, nevertheless, this hindrance effects were not observable, and was over weighted by the brilliant conductivity afforded by the SnO_2_ (see Table 2, S0&S7.5). Detailed reasons for the synergistic effects are still to be exploited, but the special structure and the ultra-small size of the SnO_2_ NCs could have a great impact on these effects. This feature would surely benefit the photon-electron conversion when the film was used as photo-anodes of the DSSCs.

### Photovoltaic study on the DSSCs

**Figure 4:**
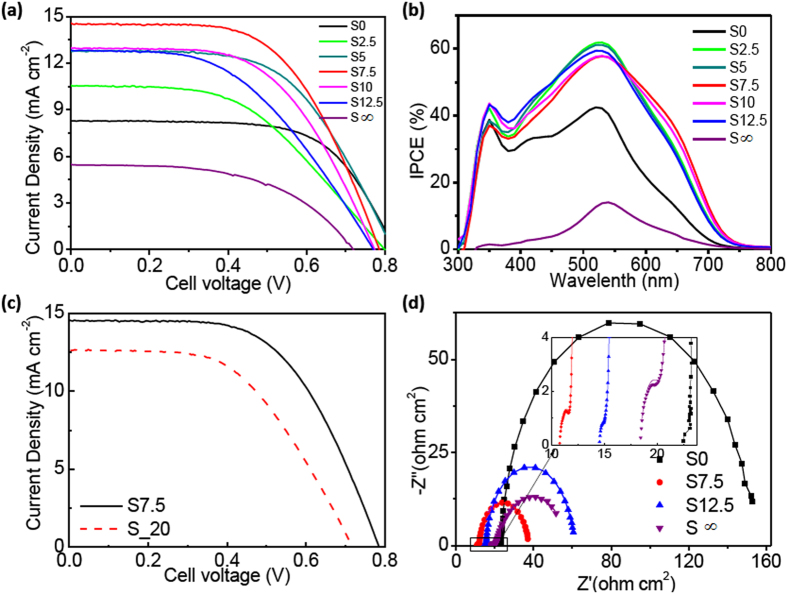
The *J*-*V* curves (**a**) and IPCE plots (**b**) of the DSSCs constructed with the SnO_2_/TiO_2_ photo-anodes (film S0, S2.5, S5, S7.5, S10, S12.5 and S∞); (**c**) The *J*-*V* curves of DSSCs constructed with P25 + S_20 and film S7.5; (**d**) The Nyquist plots of DSCs constructed with photo-anode films of S0, S7.5, S12.5, and S∞.

To study the photovoltaic properties of the DSSCs fabricated with the SnO_2_/TiO_2_ composites, the characteristic of *J*-*V* curves and the IPCE plots were recorded (Fig. 4a,b). Parameters such as *V*_OC_, short-circuit current density (*J*_SC_), fill factor (FF), *η* and the maximum values of IPCE are summarized in Table 3. Although the incorporation of SnO_2_ caused a slight decrease in the *V*_OC_ (0.82 → 0.79 V for films S0 → S7.5), the *J*_*SC*_ was increased greatly from 8.2 to 15.4 mA cm^−2^. The *η* value was found to be improved from 4.4% to 6.7% after the incorporation of SnO_2_ NCs. The highest *η* (6.7%) was found in case of film S7.5, and this value is even higher than the photo-anode constructed by the TiO_2_-coated SnO_2_ hollow microspheres (MHSs) as reported (*η* = 5.56%) by Cao *et al*.^22^. This comparison was carefully done because one may doubt on the experimental errors that would be caused by factors such as thickness of films, electrolytes, amounts of N719, counter electrodes, different batches of samples, and so on. Herein, we found the *η* value (2.1%) in our sample S∞ (the pure SnO_2_ NCs) is equivalent to that (*η* = 1.4%) of MHSs by Cao *et al*.^22^. Therefore, we think the comparison is trustable because all our results were obtained in the same batch of experiments. For other comparison, we have fabricated DSSCs by using the hydrothermally synthesized SnO_2_ (S_20) as dopants for the photo-anodes. The ratio of SnO_2_/TiO_2_ was also set at 7.5% in this composite film. As shown in Fig. 4c, the values of *V*_OC_, *J*_SC_ and *η* for the film S_20 were lower than those of fabricated with the hot-bubbling synthesized SnO_2_ NCs. Therefore, these superior performances illustrate that better photovoltaic properties can be achieved by the incorporation of SnO_2_ NCs.

To understand the interfacial reactions of photo-excited electrons and resistance across the DSSCs, we conducted the electrochemical impedance spectroscopic (EIS) measurements on the fabricated DSSCs in darkness. The bias voltage was set at the V_OC_, and the frequency ranges from 0.1 Hz to 1 MHz. The Nyquist plots are presented in Fig. 4d. All plots exhibit double semicircles of which a small-radius semicircle was found in the high-frequency region ( > 1 kHz, see the inset of Fig. 4d) and a larger one within 100–1.0 Hz. An equivalent circuit (Fig. S1) has been used to fit the Nyquist plots. The circuit and the impedance parameters derived from the Nyquist plots are listed in Table S1 (see the supporting information). The *R*_S_ representing the Ohmic serial resistance, can be read directly from the onset of the first semicircle^43^. The derived resistance *R*_1_ corresponds to the charge transfer resistance across the counter-electrode/electrolyte interface (smaller semicircle), and another part *R*_*2*_ is ascribed to the resistance between the oxide/electrolyte interface and the photo-anode film (larger semicircle)^34^,^35^. As can be found, all the *R*_*s*_ are of the same magnitude order, i.e., values range between 10.8 and 22.3 Ω cm^2^. In accordance with the photovoltaic performances of the S12.5 which is inferior to the S7.5 fabricated DSSCs, the *R*_*s*_ of the former was found larger than the later. A possible explanation for the low *R*_*s*_ in the S7.5 photo-anode could be related to the better Ohmic contact with SnO_2_ NCs at the percentage of 7.5%. For the charge transfer resistance, we got smaller *R*_*2*_ from the SnO_2_/TiO_2_ composite (e.g., 25.3 Ω cm^2^ for S7.5) as compared with the P25 film (129.4 Ω cm^2^), but the *R*_1_ is equivalent. The smaller *R*_2_ implies that the incorporation of SnO_2_ accelerates the charge transfer from the electrolyte to the photo-anode. The accelerated transfer can be resorted to the improvement in the full access of the electrolyte and the better charge mobility provide by the SnO_2_ NCs. Probably the special structures and the larger surface area can also be additional reasons. However, more incorporation of SnO_2_ NCs was found not to benefit the photovoltaic performance due to the increment of interfacial recombinations^20^. The equivalent *R*_1_ (see Table S1, S0–S∞) means that the resistance of charge transfer at the counter electrodes (Pt) are the same. Results from the EIS measurements were found to be consistent with the 4-points resistivity characterizations, which further validates the incorporation of SnO_2_ being favourable to improve the photovoltaic performances. The optimum percentage of SnO_2_ was found to be 7.5%, and the corresponding cell yields the highest *η* up to 6.7%.

**Figure 5:**
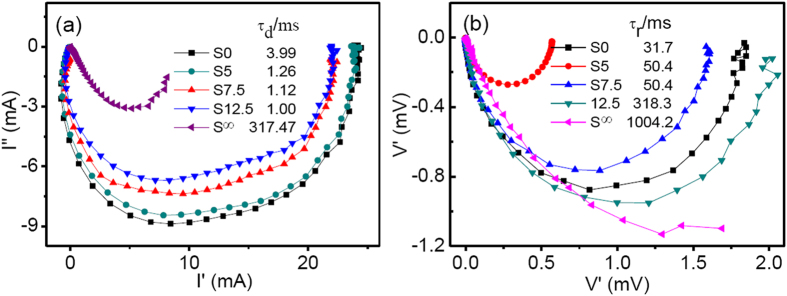
(**a**) The IMPS complex plane plots and (**b**) The IMVS complex plane plots of the composite photo-electrode for the films of S0, S5, S7.5, S12.5 and S∞.

The electron transport and recombination at the interface of photo-anode were further studied by techniques of the intensity-modulated photovoltage and photocurrent spectroscopies (IMVS and IMPS)^44^,^45^,^46^. Figure 5a shows the IMPS plots recorded under illumination of 470 nm. The electron transport time *τ*_d_, which demonstrates the average time intervals from the generation to collection of electrons, can be derived from the equation *τ*_d_ = 1/(2π*f*_min_), where *f*_min_ is the frequency of the minimum point in the IMPS semicircle. The *τ*_d_ for films S0, S5, S7.5, S12.5 and S∞ were evaluated to be 3.99, 1.26, 1.12, 1.00 and 317.47 ms. It was found that the *τ*_d_ was greatly reduced when the SnO_2_ NCs were incorporated, which means the shorter time period for the photo-electrons to reach the FTO substrate. The recombination time constant (*τ*_r_) for photo-electrons and ions of I_3_^−^ or other redox couples in the electrolyte can be derived from the IMVS plots. The *τ*_r_ was obtained according to the equation *τ*_r_ = 1/(2π*f*_max_)^36^,^37^, where *f*_max_ is the frequency of the maximum point in the IMVS semicircle. As listed in Table 4, the *τ*_r_ for the SnO_2_/TiO_2_ composite film becomes longer due to the SnO_2_ NCs, which indicates that less recombination occurs at the interface of oxide/electrolyte on the basis that the same thickness for the photo-anodes are used. The charge collection efficiency (*η*_cc_) was obtained from the relation of *η*_cc_ = 1−*τ*_d_/*τ*_r_^37^. The maximum *η*_cc_ (99.7%) was obtained in the film of S12.5, and data is only a slightly higher than S7.5 (*η*_cc_ = 98.0). Thus the maximum *η* was found in film of S7.5. Base on the above analyses, we deduced that more percentages of SnO_2_ would accelerate the electron transport, and alleviate the interface charge recombination. However, the *V*_OC_ decreases notably if more SnO_2_ NCs were use, which would result in reduction of the photo-to-electron conversion. Therefore, the optimal doping amount of SnO_2_ NCs should be set at a certain amount.

## Discussions

**Figure 6:**
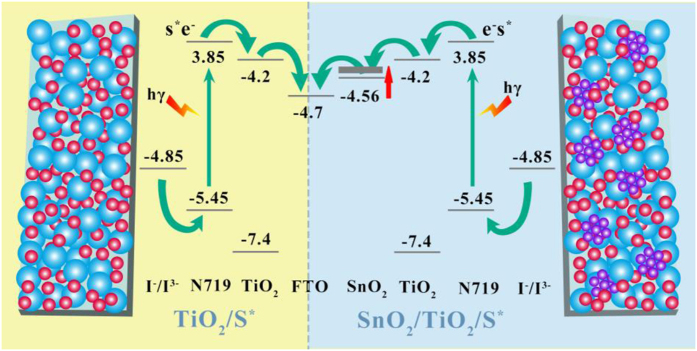
Schematic diagrams of the band alignments proposed at interfaces of oxide/dye/electrolyte for the pure TiO_2_ (left panel) and TiO_2_/SnO_2_ photo-anodes (right panel).

It is generally accepted that the decrement in the *V*_OC_ was mainly determined by the two factors^1^,^2^,^3^: (1) the conduction band shifts to more positive values because of the SnO_2_ incorporation; (2) the photo-electrons recombine with the redox couples in the electrolytes. Herein, we conducted experiments to verify these two factors and have tried to avoid the disadvantages. The first disadvantage can be avoided by decreasing the size of SnO_2_^39^. As is known, the band gaps can be enlarged by decreasing the size of a crystal. Due to the effective mass of electrons in SnO_2_ is much lighter than those of holes (*m*_e_^*^ = 0.275 m_e_, *m*_h_^*^ = 10*m*_e_^*^, *m*_e_ = 9.11 × 10^−31^ kg), the CBM is raised prominently^28^,^38^, which uplifts the Fermi level of the semiconductor electrode^41^. The position of CBM in bulk SnO_2_ is located at −4.56 V vs. vacuum^42^. According to the semi-empirical pseudo-potential method (SEPM) calculations^43^, the CBM value is uplifted to −4.34 V vs. vacuum when the sized of SnO_2_ NCs is 3.4 nm. The position lies between the CBM of TiO_2_ and the FTO (InSnO_x_ particles) electrode, which would enhance the *V*_*OC*_ and facilitate the electron transfer as well as the photo-electron injection. For a better understanding, we present a schematic diagram for the electron band alignments at the interface (Fig. 6). Based on this indication, the *V*_OC_ of the DSSCs will be enlarged as compared to the bulk SnO_2_ or micro-sized SnO_2_^27^. The second disadvantage is undertaken by reducing the trapping states. Herein, we managed to remove the trapping states by improving the crystallinity of the SnO_2_, and this was realized by utilizing a high-temperature synthesis. Due to the ultra-small SnO_2_ inserted among the P25 particles, full access of the semiconductor particles was realized^16^,^27^. This special structures of networks would surely provide efficient charge transfer pathways because of the high mobility in SnO_2_ NCs. The better conductivity of the composite films were confirmed by the 4-points conductivity and the EIS measurements. The diffusion lengths of electron (*L*_n_ = *d*(*τ*_r_/2.35*τ*_d_)1/2, *d* is the film thickness) were also calculated, which represents the average travel distance of electrons^44^. The estimated values of *L*_n_ as listed in Table 4 showed that the electron diffusion lengths were greatly lengthened due to the presence of SnO_2_. Moreover, the finding of more dye molecules adsorbed on the composite film would provide more chances for photo-electron generation, which has been testified by enhancements in the IPCE values (Fig. 4b). Therefore, the incorporation of ultra-small SnO_2_ NCs has advantages in fabricating DSSCs. The hot-bubbling synthesis can fulfil this task in preparing such ultra-small particles.

In summary, we have demonstrated the construction of highly efficient photoconversion DSSCs by using the hot-bubbling synthesized SnO_2_ NCs. The SnO_2_ NCs are of ultra-small sizes (~3.4 nm). Due to the quantum size effects the CBM of SnO_2_ is uplifted, which alleviates the decrement in *V*_OC_. The SnO_2_ NCs are of high crystallinity, and this would help to suppress the interfacial recombination of the photo-electrons. The composite film of SnO_2_/TiO_2_ exhibits a higher photo-current density and photo-conversion efficiency as compared to the hydrothermally synthesized SnO_2_/TiO_2_ photo-anodes. The higher mobility and concentration of the charge carriers in SnO_2_ were confirmed to improve the current density of the DSSCs. Compared to the pure TiO_2_ (P25) photo-anode, the transport time (*τ*_d_) for electrons injection to the FTO substrates was greatly reduced. The optimal percentage of SnO_2_ NCs was found at 7.5%, and the fabricated DSSC delivers a higher *η* of 6.7%, which is 1.52 times as that as that of pure TiO_2_ based photo-anode.

## Methods

### Materials and chemicals

The TiO_2_ (P25) (Degussa product with a mean size ~25 nm and a BET surface area of 45.4 m^2^/g) particles were used in this research. The tin (IV) chloride pentahydrate (SnCl_4_·5H_2_O, A.R.), oleylamine (OLA, 80–90% C18 content), oleic acid (OA), 1-octadecene (ODE, 90%), stannous sulfate (SnSO_4_), sodium citrate (Na_3_C_6_H_5_O_7_·2H_2_O), were obtained from Sigma Aldrich. The dye sensitizer—cis-bis (isothiocyanato) bis (2, 20-bipyridyl-4, 40-dicarboxylato) ruthenium (bis-tertrabutylammonium) (N719) was purchased from Solaronix SA, Switzerland.

### Synthesis of ~3.4 nm SnO_2_ NCs and the ~20 nm SnO_2_

For a typical hot-bubbling reaction, the synthesis of colloidal SnO_2_ NCs were performed in a three-neck flask linked with the Schlenk line^31^. Reagents including new-prepared SnO·xH_2_O (1.0 mmol, x = 1.0), OA (5.2 mL, 4.0 mmol), OLA (2.0 mL) and ODE (20 mL) were loaded in a three-neck flask. The synthesis of ~3.4 nm SnO_2_ NCs was undertaken as reported^31^. Before the synthesis all the volatile substances were removed by vacuum distillation (~0.01 bar) at 100 °C in order to purify the solvent. Under atmosphere of N_2_ the mixture solution was then heated up to 220 °C until a clear and colourless solution was obtained. A flow of air was bubbled at temperatures of 280~320 °C in order to get uniform sized NCs. The air was bubbled through a glass delivery tube. The tube has multiple pinholes (D = 0.5 mm) at one end which was exposed to the hot solutions. Samples were purified by precipitation employing toluene as solvent and isopropanol/methanol (1.0, v/v) as non-solvent. The obtained ~3.4 nm SnO_2_ samples were dried in vacuum at 60 °C.

For a control experiment we also synthesized the SnO_2_ particles (~20 nm) via a hydrothermal pathway^27^. Briefly, the ligand Na_3_C_6_H_5_O_7_·2H_2_O (4.412 g, 15.0 mmol) dissolved in a solution of 10.0 ml ethanol and 90.0 ml deionized water was mixed with SnSO_4_ (1.076 g, 5.0 mmol). After homogenization the dispersion was transferred to a Teflon-lined autoclave. The dispersion was maintained at 180 °C for 12 hrs. The product in form of light yellow precipitates was collected by centrifugation, washed with distilled water/ethanol. The SnO_2_ microspheres were obtained after drying in vacuum at 70 °C for 24 hrs. The powders were annealed in air at 450 °C for 2.0 hrs. This sample (termed as S_20) was also used as the photo-anode material.

### DSSCs fabrication

To fabricate the photo-anodes of the DSSCs, pastes of TiO_2_ (P25) and SnO_2_ were prepared. Into an agate mortar 1.0 g P25 powders and a certain amount of SnO_2_ NCs were loaded, and the mixture was grinded for ~0.5 hrs before the addition of acetic acid (99.7%, 100 μl), deionized water (50 μl) and ethanol (50 ml). The suspension was further grinded for another 0.5 hrs, and was then transferred to a flask for sonication (~1.0 hrs). A mixture of terpineol (3.5 g) and ethyl cellulose (0.5 g) was added, and the final homogeneous paste was prepared by the repeated procedures of magnetic stirring and sonication. The excessive ethanol was removed by rotary-evaporator in a round-bottomed flask until a percentage of ethanol (~15% *wt.*) was left. In this experiment different ratios of SnO_2_ were added to prepared the composites of SnO_2_/TiO_2_, i.e., percentages of SnO_2_/TiO_2_ = 0%, 2.5%, 5%, 7.5%, 10%, 12.5% and ∞% were achieved in the paste. The sample of ∞% denotes the pure SnO_2_ NCs. These samples were labelled as S0, S2.5, S5, S7.5, S10, S12.5 and S∞. For comparison, the S_20 nm was also employed to prepare a paste in a similar procedure.

Films of photo-anodes were prepared by applying pastes of S0–S∞ and S_20 on an electric conducting glass plate. The fluorine-doped tin oxide (FTO, Geao Co., Wuhan, China) glass plates were used as the electric conductive substrates. A layer of mixture film (0.5 × 0.5 cm^2^) was fabricated on a FTO glass via the method of screen-printing. The film was then dried at an oven at 125 °C for 6.0 min. Subsequently the film was annealed at 500 °C for ~30 min to generate the porous structure. The porous films in thickness of ~8.0 *μ*m were further coated with another layer of TiO_2_ by dipping in a 0.05 M TiCl_4_ aqueous solution for 30 min. After drying, the plate was sintered in air for 30 min at 500 °C. The electrodes was sensitized with dye molecules by immersing in a 50 mM N719 solution of acetonitrile/t-butyl alcohol (v:v = 1:1). The adsorption time is 24 hrs, and the temperature is room temperature (~25 °C). Finally, the electrodes were washed with ethanol and dried in vacuum (~0.01 mbar). The DSSCs were fabricated with a sensitized photo-anode, a platinized (Pt) counter electrode and the electrolyte. The DSSCs were sealed with a hot-molten gasket. The electrolyte consists of lithium iodide (LiI, 0.045 M), iodide (I_2_, 0.032 M), 4-ter-btylpyridine (TBP, 0.5 M), guanidinium thiocyanale (0.1 M), 1-butyl-3-methylimidazolium iodide (BMII, 0.6 M) and acetonitrile/valeronitrile (85/15 vol %), which is similar to our previous reports^45^.

### Characterizations

Phases of materials were determined by the X-ray diffraction (XRD, Rigaku Co., Japan) using Cu Kα (*λ* = 0.15418 nm) as the irradiation. The morphology and microstructures were studied by the field emission scanning electron microscopy (FESEM, SU8020) and transmission electron microscopy (TEM, JEM-2100F). For the Fourier transform infrared (FT-IR) analyses, the spectrum was collected on the BRUKER TENSOR 27 instrument. The surface area and pore size distribution were obtained from a MICROMERITICS-ASAP 2010 unit, and the sample was activated in the N_2_ atmosphere (t = 2.0 hrs; P = 10.0 bar; T = 423 K). The electrical properties were tested by means of 4-point probe resistivity and Hall-effect measurements at T = 298 K by using an ET9107 system. The applied magnetic field was 0.322 T. Films with dimensions of 1.0 × 1.0 cm^2^ were linked by a four-Au-tip in the Van der Pauw geometry. The film thickness was determined to be ~5.0 *μ*m by a step profiler (ET150). This technique allows the determination of the resistivity, the charge carrier concentration and the mobility. The photocurrent density-voltage (*J*-*V*) characteristics of the DSSCs with an active area of 0.25 cm^2^ were carried out under AM 1.5 (100 mW cm^−2^) illumination, which was provided by a solar simulator (Oriel Sol 3A Solar Simulator, USA) liked with a Keithley digital source meter (Type 2400). The incident photon-to-current conversion efficiency (IPCE) plotted as a function of illumination wavelength, were recorded on a QTest Station 1000 ADI system (Crowntech, Inc.) equipped with a 300 W Xe lamp. The measurements were carried out by using a monochromator, assisted by an automatic filter wheel. Electrochemical impedance spectroscopic (EIS) measurements were recorded on Autolab320N electrochemical workstation. The frequency range explored was from 100 kHz to 1 Hz at a set potential of 0.78 V. The dynamic measurements of IMVS and IMPS were also recorded on the same potentiostat but linked with an intensity modulated blue LED light (470 nm).

## Additional Information

**How to cite this article**: Mao, X. *et al*. High Efficiency Dye-sensitized Solar Cells Constructed with Composites of TiO_2_ and the Hot-bubbling Synthesized Ultra-Small SnO_2_ Nanocrystals. *Sci. Rep.* **6**, 19390; doi: 10.1038/srep19390 (2016).

## Supplementary Material

Supplementary Information

## Figures and Tables

**Table 1:** The surface density of adsorbed dye molecules N719 and results derived from the N_2_ adsorption-desorption isotherms of the SnO_2_/TiO_2_ films.

Film	Weight ratio of SnO_2_/TiO_2_ (%)	Adsorbed N719 (10^−7^ mol cm^−2^)	Surface Area (m^2^ g^−1^)	Pore Volume^a^ (cm^3^ g^−1^)	Average Pore Size^b^ (nm)
S0	0.0	1.70	45.4	0.22	28.4
S7.5	7.5	1.90	53.0	0.27	20.5
S12.5	12.5	1.57	53.6	0.25	18.7
S∞	∞	0.71	64.8	0.06	3.9

^a^The total pore volume was evaluated for a P/P_0_ ratio of 0.99.

^b^Adsorption average pore width (4 v/A by the BET method).

**Table 2:** Resistivity, carrier concentrations and Hall mobility derived from the 4-point probe resistivity/Hall-effect measurements.

Film	Resistivity (Ω cm)	Hall coefficient (cm^3^ C^−1^)	Carrier concentration (cm^−3^)	Hall mobility (cm^2^ V^−1^ s^−1^)	P/N type
S0	4.57	9.1 × 10^3^	6.86 × 10^14^	6.48	N
S7.5	3.74 × 10^−2^	2.43 × 10^−1^	2.57 × 10^19^	1.98 × 10^3^	N
S12.5	5.87 × 10^−2^	6.99 × 10^−1^	8.93 × 10^18^	1.19 × 10^2^	N
S∞	7.14 × 10^−2^	3.57 × 10^−1^	1.75 × 10^17^	4.99 × 10^2^	N
S_20	4.71 × 10^−3^	1.81 × 10^−1^	3.45 × 10^18^	3.83 × 10^2^	N

**Table 3:** Photovoltaic parameters of DSSCs constructed with different photo-anodes (S0–S∞, and S_20).

Film	*V*_OC_ (V)	*J*_SC_ (mA cm^−2^)	FF	*η* (%)	IPCE (%)
S0	0.82	8.34	0.64	4.4	42.5
S2.5	0.80	10.60	0.49	4.5	61.9
S5	0.80	12.79	0.57	6.0	59.4
S7.5	0.79	14.53	0.58	6.7	61.2
S10	0.78	13.01	0.56	5.6	57.5
S12.5	0.77	12.85	0.48	4.8	57.9
S∞	0.72	6.80	0.53	2.1	14.1
S_20	0.72	12.45	0.51	4.8	56.4

**Table 4:** Photovoltaic parameters of the electron transport time (*τ*_d_), the recombination time (*τ*_r_) and the collection efficiency derived from measurements of IMPS and IMVS.

Film	τ_r_ (ms)	τ_d_ (ms)	*η*_*cc*_ (%)	*L*_n_ (*μ*m)
S0	31.7	3.99	87.4	14.7
S5	50.4	1.26	97.5	33.0
S7.5	50.4	1.12	98.0	35.0
S12.5	318.3	1.00	99.7	93.1
S∞	1004.2	317.47	68.4	9.28
