# Supplementary material for: High Efficiency Dye-sensitized Solar Cells Constructed with Composites of TiO2 and the Hot-bubbling Synthesized Ultra-Small SnO2 Nanocrystals
Source: Sci Rep. 2016 Jan 13;6:19390. doi: 10.1038/srep19390 (PMC4725357; doi:10.1038/srep19390)
Supplement: Supplementary Information [file srep19390-s1.pdf]

High Efficiency Dye-sensitized Solar Cells Constructed with Composites of TiO2 and the Hot-bubbling Synthesized Ultra-Small SnO2 Nanocrystals

Xiaoli Mao,[1] Ru Zhou,[1] Shouwei Zhang,[1] Liping Ding,[2] Lei Wan,[1] Shengxian Qin,[1] Zhesheng Chen,[3] Jinzhang Xu,[1] and Shiding Miao *[2]

[a] School of Electronic Science and Applied Physics, Hefei University of Technology (HFUT), Hefei 230009, China

[b] School of Chemistry and Chemical Engineering, HFUT, Hefei, 230009, China

[c] Institut de Minéralogie, de Physique des Matériaux, et de Cosmochimie (IMPMC), Sorbonne Universités - UPMC Univ Paris 06, Paris 75005, France.

* Corresponding author.

Tel: 86-551-62901126; Fax: 86-551-62901115, E-mail: miaosd@iccas.ac.cn (S. Miao), xujz@hfut.edu.cn (J. Xu).

1. Equivalent circuit for fitting the Nyquist plots

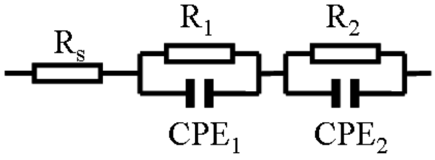

Figure S1 The equivalent circuit for fitting the Nyquist plots (Figure 4d, in the main manuscript).

Table S1 Impedance parameters derived from the Nyquist plots in Figure 4d.

Film	R_s (Ω cm^2)	R_1 (Ω cm^2)	R_2 (Ω cm^2)
S0	22.3	0.9	129.4
S7.5	10.8	1.1	25.3
S12.5	14.5	1.0	45.2
S∞	18.3	2.3	37.4
